# A framework for remotely enabled co-design with young people: its development and application with neurodiverse children and their caregivers

**DOI:** 10.3389/fpsyt.2024.1432620

**Published:** 2024-08-16

**Authors:** Anna Charlotte Morris, Stephen Douch, Teodora Popnikolova, Chris McGinley, Faith Matcham, Edmund Sonuga-Barke, Johnny Downs

**Affiliations:** ^1^ CAMHS Digital Lab, Department of Child and Adolescent Psychiatry, Institute of Psychiatry Psychology and Neuroscience, King’s College London and South London and Maudsley National Health Service (NHS) Foundation Trust, London, United Kingdom; ^2^ Age and Diversity, Helen Hamlyn Centre for Design, Royal College of Arts, London, United Kingdom; ^3^ School of Psychology, University of Sussex, Brighton and Hove, United Kingdom; ^4^ Department of Child and Adolescent Psychiatry, Institute of Psychiatry, Psychology and Neuroscience, King’s College London, London, United Kingdom

**Keywords:** remote co-design, inclusive design, qualitative methods, young people, neurodevelopmental conditions, ADHD

## Abstract

**Introduction:**

This paper describes an innovative Framework for Remotely Enabled Co-Design with Young people (FREDY), which details an adaptable four-stage process for generating design concepts with children and other key stakeholders in a naturalistic and inclusive way.

**Methods:**

Recommendations from existing patient engagement and design methodologies were combined to provide research teams with procedures to capture and analyse end-user requirements rapidly. Resulting insights were applied through iterative design cycles to achieve accelerated and user-driven innovation.

**Results:**

Applying this framework with neurodiverse children within the context of healthcare, shows how creative design methods can give rise to new opportunities for co-creating across diverse geographies, abilities, and backgrounds as well as strengthen co-designer approval of the co-design process and resulting product.

**Discussion:**

We summarise key learnings and principles for fostering trust and sustaining participation with remote activities, and facilitating stakeholder design input through continuous collaboration, as well as highlight the potential benefits and challenges of utilising FREDY with neurotypical populations.

## Introduction

Practices in healthcare systems must be continually adapted and improved to provide the best care and outcomes for their patients (1, 2). Failure to consider or directly consult patients during the design of novel interventions or systems has resulted in the development of products that do not fully encapsulate end-users’ needs, human context, or fallibility. This has led to a pronounced intervention implementation gap (3–5) and a substantial waste of health research resources (6). In the last two decades, there has been a major shift towards patient and public involvement (PPI) health research, which has been increasingly acknowledged and mandated by policymakers, funding bodies, patient communities, and governmental initiatives both in the UK and globally (7). With PPI on the rise, the literature base detailing the methodology and practical techniques for operationalising patient partnership is expanding (8–10). One approach which has been used to address PPI requirements in healthcare and guide researchers in developing more patient-centred, accessible, and usable solutions is inclusive design (11). Inclusive design principles state that capability levels differ across the population and seek to develop products that accommodate this diversity by considering the needs and characteristics of the widest possible user group (12, 13). This is achieved through designing with ‘unheard voices’ - bringing people with lived experience who are often excluded into the design process early on. In this regard, inclusive design aims to facilitate change by engineering products which are functional and empowering to all (12).

Co-design is a key method used in inclusive design. This practice combines insights from end-users as ‘experts of their experiences’ and other key stakeholders with professional input from qualified designers at the formative design stage to develop credible and acceptable solutions which enhance real-world application and use (14). Importantly, this approach has been credited with shifting the design rhetoric from a focus on designing ‘for’ to designing ‘with’ end-users as equal partners in a creative collaborative process (15). Co-design includes participants throughout the design process to guide the development of products and services, ensuring all voices and perspectives are valued. Co-design approaches are known to enhance innovation benefits (16) and empower end-users by increasing awareness and knowledge about their condition, enabling personal contribution to their health and well-being (7, 17). This collaborative approach also increases the likelihood of product adoption and sustained engagement (18). Co-design has been successfully applied to a breadth of health-oriented sectors, including health services (19, 20), health technology (21), and quality improvement (22, 23) and has enabled meaningful participation of vulnerable populations (24–27), including physically disabled (28–30) and neurodiverse children (31–33).

To date, co-design research in healthcare has generally favoured in-person engagements like face-to-face workshops, interviews and focus groups for data acquisition (34). However, the COVID-19 pandemic necessitated a rapid move to online co-design owing to severe disruption caused by social distancing restrictions, which prohibited assembling participants in a shared physical space (35–37). Despite the return of some research practices post-pandemic, remote data acquisition remains popular. However, well-defined guidelines on how to best conduct traditional co-located research virtually are still needed. Facilitating the continuity of remote co-design in healthcare in a virtual context is crucial to enable scientific progress and inform clinical practice while maintaining data quality and integrity (38). Shifting to more flexible participatory research aligns with broader calls, for more adaptable ways of working with end-users to accommodate specific project constraints and emerging design spaces (39). This is especially true for healthcare co-design, which demonstrates substantial variation in approaches and extent of service-user engagement (40, 41). Moreover, web-based co-design has proven useful for conducting co-design with adults and children (42–45). To our knowledge, the feasibility of applying these translational techniques remains unexplored for healthcare product and intervention innovation with children who have attention deficit hyperactivity disorder (ADHD), a population with diverse needs that may present distinct challenges to ensuring inclusivity (31).

The current paper describes our search for and subsequent creation of a novel engagement framework for remote co-design using an inclusive design approach; Framework for Remotely Enabled Co-Design with Young people (FREDY). We use our work, the Paediatric Actigraphy Clinical Evaluation System (PACES) wearable activity tracker as a case vignette to illustrate framework implementation, documenting the tools and techniques used to facilitate virtual co-design in the context of childhood ADHD.

## Materials and methods

### Project overview

This project represents the initial work stream of a larger five-year funded research programme (https://fundingawards.nihr.ac.uk/award/CS-2018-18-ST2-014). The overarching aim of this programme is to investigate if an end-user-designed digital health monitoring system can improve the quality of information used to monitor treatment effectiveness and safety in childhood ADHD treatment beyond subjective ADHD rating scales. The objective of the current workstream was to co-design and manufacture a novel, validated, low-cost (under £20 per unit) wearable actigraphy device to monitor free-living physical activity patterns in children with ADHD aged between five and 11 years old which is acceptable to this cohort. This programme of works highlights three key challenges that make it suitable for testing remote codesign methods.

#### Clinical challenge

In current clinical practice within the ADHD treatment and diagnostic pathway, questionnaires are completed by multiple informants; these afford low inter-rater agreement (46) and are susceptible to nocebo and placebo effects, which can hinder clinical decision-making. Accordingly, there is a need for more robust and objective information on the treatment effects amongst children taking medication to treat symptoms to supplement subjective methods. Actigraphy, an objective assessment of movement, is widely used in research to measure the presence and severity of ADHD symptoms and treatment side effects (47). It effectively discerns activity and sleep patterns in medicated versus unmedicated children (48, 49). Moreover, actigraphy demonstrates good predictive validity with subjective questionnaires (50, 51). Despite the nuances within ADHD diagnosis, actigraphy-derived hyperactivity is a prominent feature across both combined and inattentive subtypes (52, 53), making it a valuable tool for monitoring ADHD broadly.

#### Design challenge

The design challenge relates to the suitability and usability of a new routine monitoring tool. While numerous commercial activity tracking devices exist, they tend to be feature rich, and therefore, may be deemed too distracting to be worn in education settings (54), and could be readily lost or damaged rendering them expensive to replace (55). Most devices afford a high recharge burden, for example, Fitbit devices tend to require weekly charging for one-to-two hours, and need to be removed to do so, which can contribute to data missingness (56). Access to the algorithmic models used to process data from these devices is often not publicly available nor is there an obligation for manufacturers to publish algorithm changes which might bias or comprise the scientific validity of the resulting data (55, 57). Similarly, data settings tend to be pre-set by the manufacturer and may be too crude to detect meaningful changes in activity. Furthermore, many of the available models do not accommodate the full range of paediatric wrist sizes, often resulting in large overlaps of strap material or ill-fitting, uncomfortable devices (58). Using data collected from commercial wearable devices within routine healthcare also raises additional complexities. Implementing GDPR-complaint data storage for patient data necessitates considerable effort and resources to ensure stringent data governance frameworks and secure APIs, for example, the National Health Service (NHS) permits the use of third-party cloud-based servers storing patient data, providing the servers are located within the UK (59). Additionally, interoperability challenges ensue from needing to integrate device generated data into existing healthcare systems. Medical grade actigraphic devices also exist and have been used in research to assess physical activity in children, though these tend to be expensive (60) and once again not designed to fit primary school-aged children. Moreover, these devices are normally worn for short observation periods, as opposed to extended monitoring which may be required for biomedical monitoring (49), which may be less tolerable. Accordingly, these models are not viable for large-scale use in the NHS.

#### Methodological challenge

Before COVID-19, traditional in-person co-design methods were often favoured as an effective approach for fostering engagement, equalising power dynamics, and sustaining participation over time (15). When within-person research was prohibited due to social restrictions, the challenge became developing digital co-design strategies that could fulfil the same functions, including building and sustaining relationships online and, maintaining engagement while limiting digital fatigue, facilitating creative collaboration, consciously creating an equal distribution of power, considering participants’ skills and abilities, and ensuring accessibility (45, 60).

### Co-design team

To ensure the domain topic was fully explored from the perspective of key stakeholders with a vested interest in the product and end-users’ needs, as well as establish necessary within-team expertise, we identified, selected, and recruited co-designers using Dix and colleagues (61) stakeholder analysis model, which separates co-designers into four categories.

- Primary stakeholders – people who interact directly with the product.
- Secondary stakeholders – people who interact indirectly with the product either through product input or output.
- Tertiary stakeholders – people who are affected by the product but not via direct or indirect interaction.
- Facilitating stakeholders – people responsible for the design, development, and implementation/maintenance of the product.

#### Primary stakeholders

Voluntary sampling was used to recruit eight children and their caregivers as ‘experts through experience’ from a larger ADHD advisory group consisting of approximately 30 caregivers of children for in-depth participation in the project. Involvement from the ADHD group occurred on an *ad-hoc* basis depending on need. Children were diagnosed with ADHD within the age range of the intended PACES end-user (5–11-year-olds). To ensure adequate representation we surmised that recruitment should be proportional to the population prevalence of ADHD diagnosis between sexes and therefore recruited ADHD participants on a 4:1 male-to-female ratio. Rather than co-design with children independently, caregivers acted as co-facilitators to provide in-person support for their child during the process (62, 63).

#### Secondary stakeholders

Purposeful sampling was used to recruit three healthcare practitioners with experience treating ADHD children (two senior child psychiatrists and ADHD nurse specialists) from South London (local) and (one senior child psychiatrist) national and specialist child and adolescent mental health services. These stakeholders were identified as integral informants as health professionals are often the primary contact for families with children with ADHD. Ultimately these individuals will use information collected by the PACES device to inform clinical decisions and therefore, may have ideas around essential functionality.

#### Tertiary stakeholders

Again, purposeful sampling was used to recruit educational professionals with experience in teaching children with ADHD from London and the surrounding areas. Specifically, we spoke to one head of school, one special educational needs coordinator, and one class teacher to get a variety of viewpoints. Importantly, teachers are responsible for enforcing school fashion and accessory policy and were deemed helpful in understanding the type of wearable technologies permitted during school hours.

#### Facilitating stakeholders

This stakeholder group featured an interdisciplinary collaboration between clinical research academics from King’s College London’s Institute of Psychiatry, Psychology & Neuroscience who provided expert knowledge and an understanding of the problem area from a clinical perspective and professional designers from the Helen Hamlyn Design Centre at the Royal College of Art, a global leading institute in inclusive design provided the expertise required to develop tools to support ideation and design the end product.

The academic/clinical team included the project lead (JD), a clinical academic research fellow in child and adolescent mental health-related digital technology system development, and a consultant child psychiatrist responsible for project oversight and delivery. It also included a clinical research assistant and PhD student (AM), with a background in web-based outcome monitoring platform development and implementation in child and adolescent mental health.

The design team included the lead designer (SD) who has substantial experience in co-creation projects with children and was responsible for leading design activities; and a design managerial research fellow (CM) with over 20 years of inclusive design expertise who provided regular supervision and project consultation.

### Our approach to design

The purpose of this research was to understand how interdisciplinary teams collaborate to co-design within the constraints of remote research with children and families with ADHD. As a team, we agreed that the main challenge of this work was to determine how to best achieve creative processes in a virtual space, in a way that is engaging, non-burdensome, and inclusive for our user group (64). To collectively integrate their varied viewpoints, knowledge, and experiences into the design process (65), we had to provide suitable tools to the user group to facilitate self-expression. While there is an abundance of open book guidelines, resources and toolkits for researchers on codesign activities (The Point of Care Foundation https://www.pointofcarefoundation.org.uk/ Service Design Tools https://servicedesigntools.org/tools, IDEO https://www.ideou.com), many of the established co-design techniques and procedures typically call for a shared physical environment and materials, i.e., brainstorming, card sorting, empathy mapping and rough prototyping which may not be appropriate for codesign carried out remotely. Still, building rapport, trust, and effective communication are essential to redistribute power and foster the collaboration needed for sustainable project impact (66). Therefore, when deciding how to substitute virtual meetings for in-person meetings, we kept these needs at the forefront.

After initially conducting a focused literature review, we established that there was no suitable framework to address all these considerations and guide remote participatory design research with neurodiverse children, without needing refinement. While no one framework was sufficient as a model, we identified three main frameworks which aligned with our aim. The current framework we are proposing was developed based on adapting and combining these existing frameworks – The Double Diamond design framework (67), The Diversity for Design Framework (68), and Agile Design Methodology (69).

#### The double diamond framework

**Figure 1:**
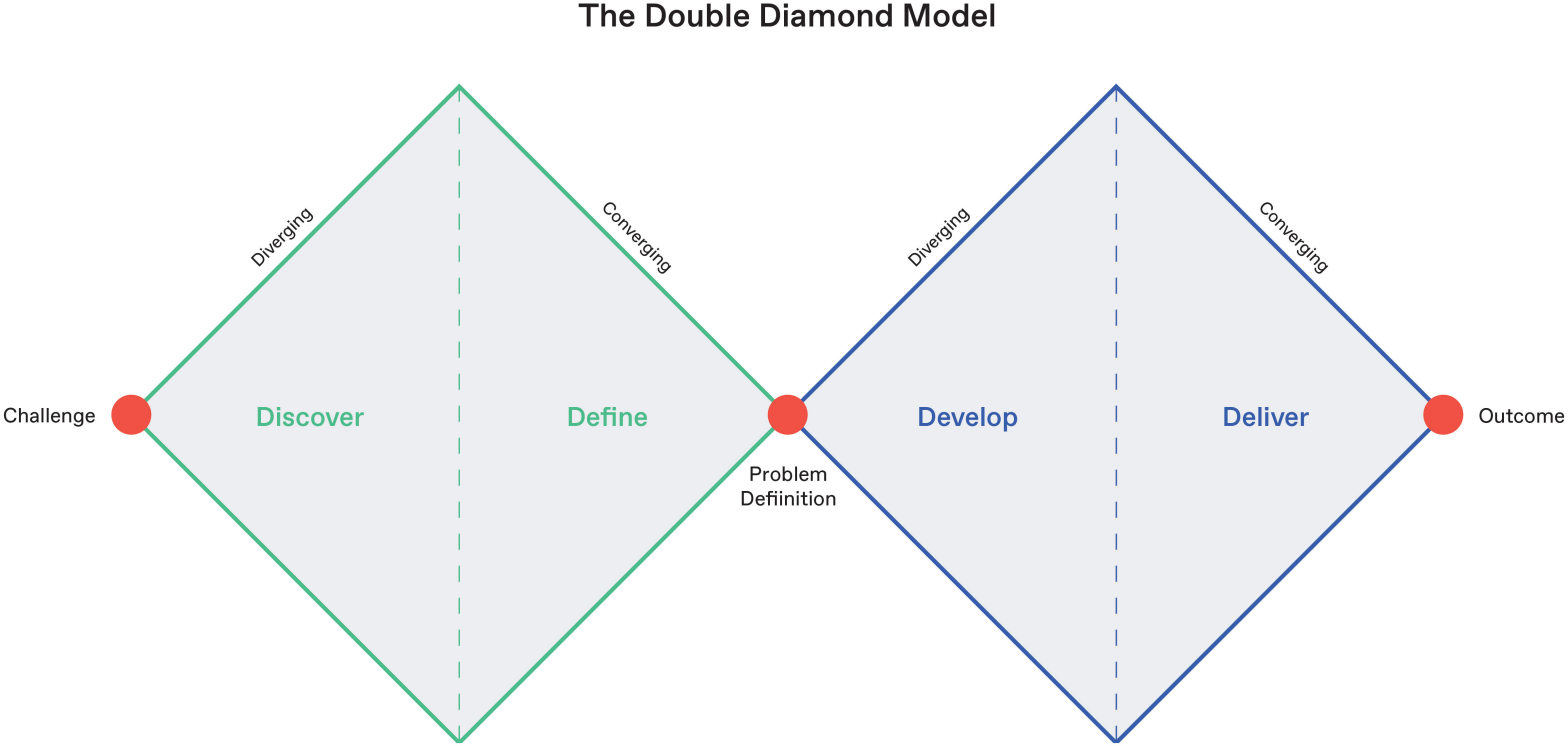
Overview of the British Design Council’s Double Diamond design framework.

The British Design Council Double Diamond (67) (**Figure 1**) is a framework for innovation which lays out a process to tackle complex design problems. At its core, the Double Diamond is an inclusive design technique that engages users in all stages of the design process to create solutions that fit their lives. The model is broken into four distinct phases: discover, understand the problem at hand, define, delineate the challenge, develop, create potential solutions, and deliver, test to find an ideal solution. Each diamond represents two equal paths of divergent-convergent enquiry, the former focusing on exploring and defining the appropriate problem and the latter exploring and defining a suitable solution. The Double Diamond model is traditionally understood as a methodology for design development to take teams from a brief to the final design. However, in the context of the PACES project, we had to adapt it to be a tool for understanding how to research and co-design with a neurodiverse childhood population. Accordingly, this tool enables us to work inclusively by considering the experiences of our user group, ensuring their needs and preferences for remote research are met.

#### Diversity for design framework

Developed by Benton et al (68), the D4D framework provides direction on how traditional co-design methods can be modified to accommodate the unique preferences of neurodiverse children. D4D advocates a supportive and strength-focused approach to designing activity structure and environment, which considers shared characteristics across neurodiverse conditions, in addition to each participant’s unique skills and abilities.

**Figure 2:**
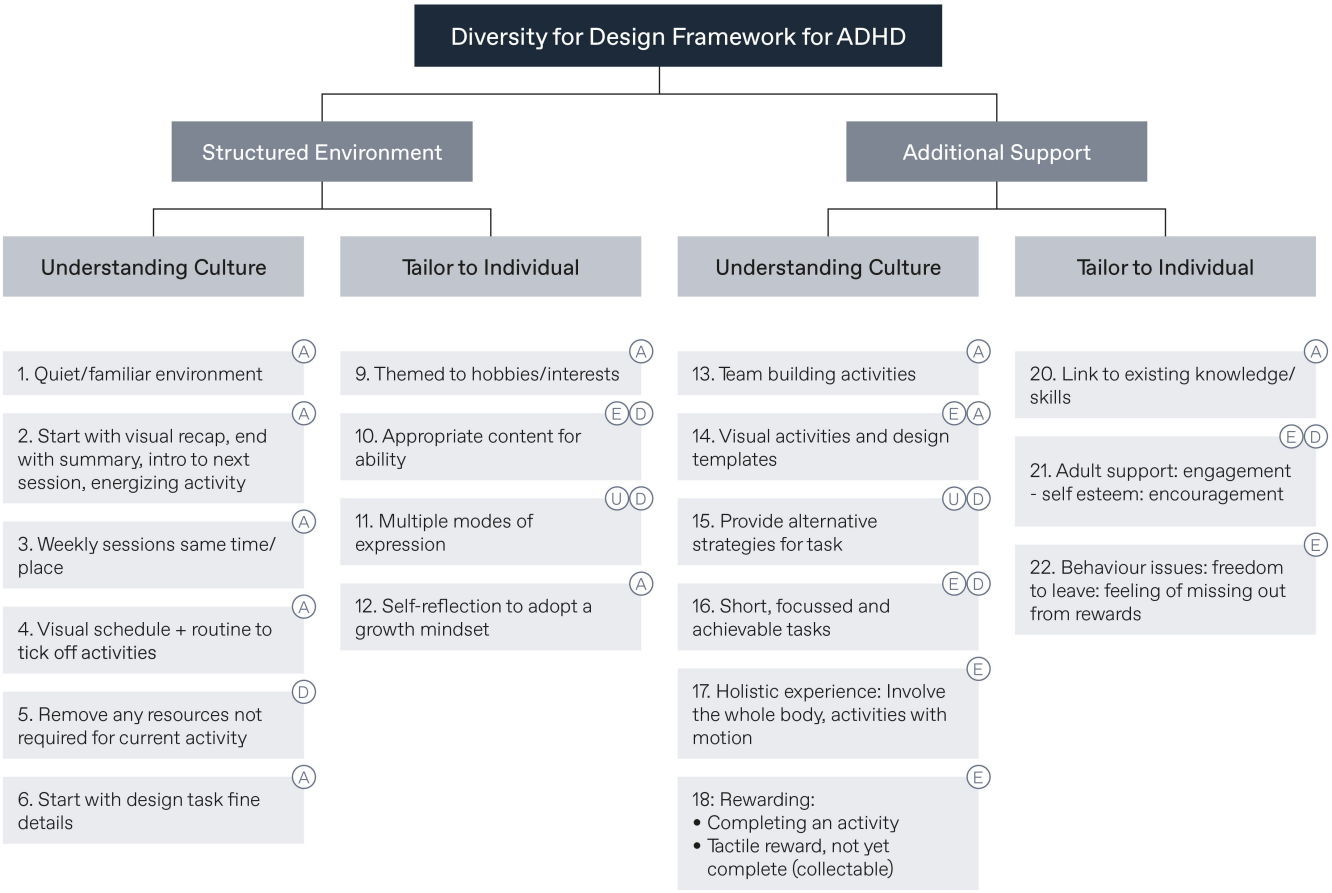
Diversity for Design (D4D) framework adapted for ADHD. Labels refer to “A” for ASD, “D” for Dyslexia, “E” for expert interviews, and “U” for the Universal Design for Learning (UDL) approach.

D4D is a broad set of guidelines that consider the need for designers to be adaptive and responsive to the inevitable influence of specific contexts and constraints. Originally trialled with case studies featuring children with autism spectrum disorder and dyslexia, Fekete and Lucero (31) have since adapted D4D for use with people who have ADHD, and it has been used by designers to co-design with this population (32). A full outline of this theoretically and practically revised framework is presented in **Figure 2**. Briefly, this revised framework recommends that designers conduct activities in quiet and familiar surroundings, ensure that sessions are short, focused, contain achievable activities, and start with a topic that would interest children, as well as provide regular breaks and rewards (33). In this study, we built further on the strategies identified in the adapted D4D for ADHD, adjusting them to suit the additional challenges posed by co-designing virtually.

### Agile design

Traditional design processes are linear, often referred to as the waterfall or gated method because progress goes in a single direction. Once decisions have been made, it is difficult or impossible to go back. Gated methods have a defined sequence: research, design, engineering, and manufacturing. They lend themselves to projects involving considerable human input, time, and budget, where iteration would be prohibitively expensive and long. This contrasts with agile design, where the process is circular, with continual refinement and change (69, 70). Originally developed to guide software development (69, 71), agile methods seek to collect and act on early feedback from the end-user to continuously improve rather than deliver a high-fidelity prototype immediately. The ability to iterate helps avoid incorrect design decisions by constantly reviewing design decisions with the end-user. Incorrect design decisions can be costly to rectify further into the design process; as Frank Lloyd Wright ‘The architect’s most effective tools are the eraser in the drafting room and the wrecking bar on the job.’ (70). Agile design is best suited for the early design phases to determine that the correct problem is being solved (the first half of the double diamond frames the correct design question i.e., problem definition). Iterative methods help clarify the problem statement and defer rigid specifications (70). Prototypes must be user-tested to refine the requirements, bringing rational changes for success, and decreasing major changes further down the line (72). As such the design team decided this would be a suitable approach for achieving product design, development, and delivery within the context of a finite grant-funded academic-industry venture.

## Results

**Figure 3:**
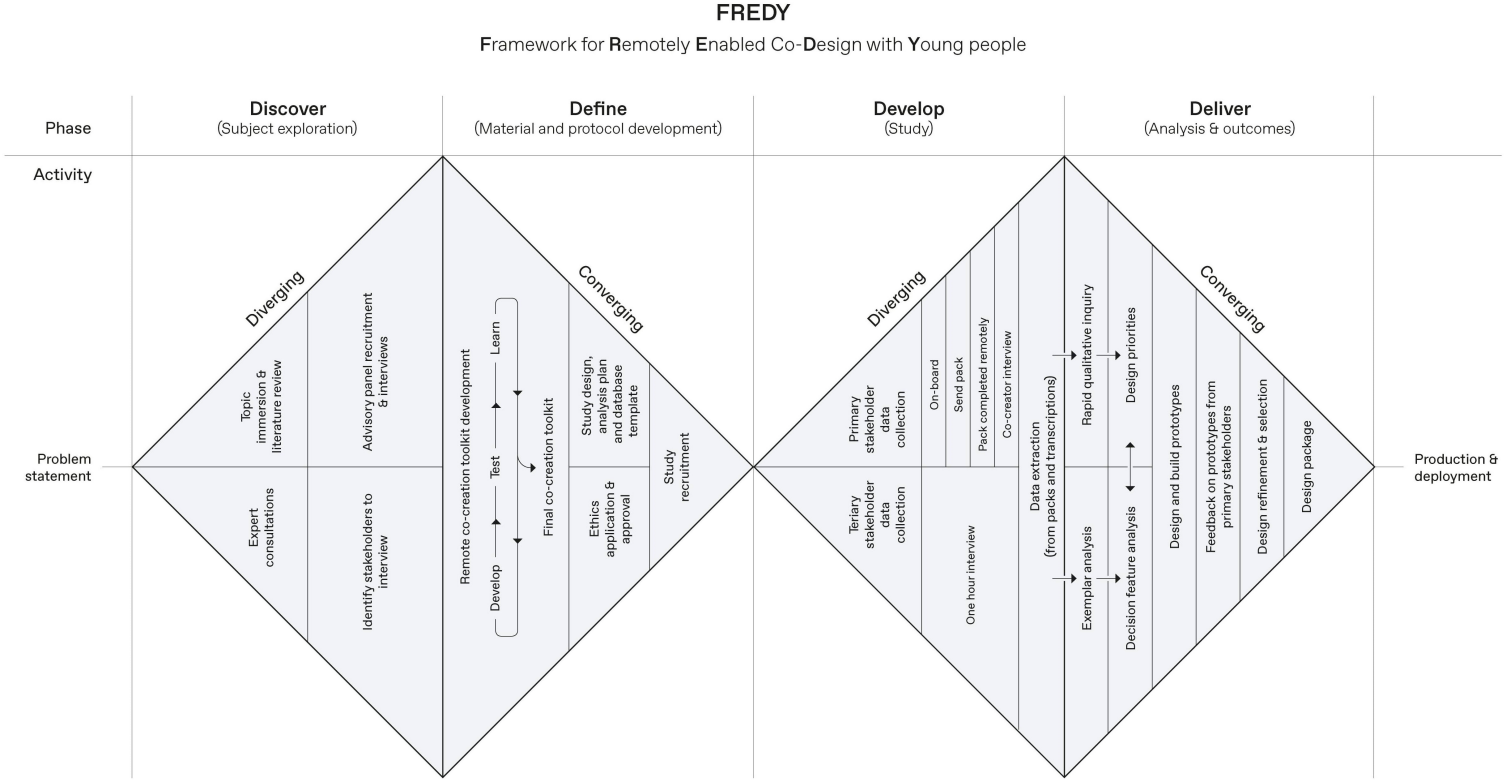
Overview of the Framework for Remotely Enabled Co-Design with Young people.

Within FREDY (**Figure 3**) modifications were made to each of the four phases of the Double Diamond framework, combining it with the D4D for ADHD framework and iterative design principles, and adapting the methodology to develop an approach for remote participatory design with neurodiverse children. Similarly, to the original Double Diamond framework, our framework consists of four phases adopting a diverging and then a converging sequence approach.

### Adapted discover phase

This phase aims to carry out formative research tasks and to identify key stakeholders. This process can involve desk research - including rapid or systematic reviews, situational analysis, or other types of context familiarisation - as well as between- and within-team round table team discussions. The purpose of these discussions is to upskill the design team members on rudimentary design principles and provide the design team members with more in-depth knowledge of the population the end product is intended for. Drivers which could promote or hinder virtual co-design implementation in the specific user group should be explored extensively with a focus on incorporating information from a variety of sources. Information resulting from these activities should inform the basis for the project’s intended approach to co-design, including but not limited to: the type of platform selected to facilitate virtual discussions i.e., video conferencing tools such as Skype, MS Teams, or Zoom, or group discussions forums e.g., Gather or Google Jamboard, and envisaged data collection aids i.e., workbooks, diaries, and creative materials. Additional advisory discussions with primary, secondary, and tertiary stakeholders can be used to further comprehend the problem area and, fundamental design constraints or features, as well as consider how the intended approach to co-design will work with the chosen population. Ethical approval for information gathering at this stage is not required as the work is purely advisory. Throughout these meetings, resulting strategies for adapting the co-design process and remote co-design material should be iteratively developed, at which point it is beneficial to meet with advisors again or wider advisory groups to sense-check the findings of this stage.

### Adapted define phase

This phase involves developing an activity pack, as well as defining a data analysis plan.

#### Activity pack

The activity pack should reflect the user group’s needs and project objectives about the type of information to be ascertained and should identify the best ways to do that. Particular attention should be paid to the user’s ethnographic infrastructure (73) – that is, the context of the users’ daily lives and how this might impact the implementation and uptake of the design solution. The activity pack should explore how the design solution and its use will fit into the end-user’s relationships (family and friends), physical surroundings (home, school, and leisure), and lifestyle factors. Collecting this type of data will provide valuable information on how external factors may influence if, when, and how the end-user engages with the product (74, 75). It should map the existing frameworks’ principles onto key sections but adapt it to remote means and consider the findings of the Discover phase. We propose an agile method of development, rapidly prototyping the pack using internal team and external advisory feedback to then learn, test, and refine the study materials through multiple iterative cycles to end up with a finished inclusive pack. Broadly, we propose that an activity pack incorporates techniques such as crafting activities (76) and creative tools which allow end-users to prototype potential design solutions (14, 76). Methods for recording individuals’ experiences outside the physical workspace i.e., ‘design probes’ (77, 78) should also be considered as a potentially helpful tool for bringing participants’ hobbies and interests into the research setting. Design probes (sometimes known as cultural probes or design provocations) refer to a collection of artefacts designed to provoke a creative and reflective response from the user regarding their past, present, and future (78, 79). This method affords a subjective representation of users’ lives which can be built upon by verbal elaboration to help foster an empathic dialogue between users and the designer. Some examples of design probes include diaries, logbooks, cameras, and open-ended questions (27, 76). The final version of the PACES activity pack can be found in **Supplementary Material 1**.

#### Data analysis plan

Next, this phase should identify a data analysis plan i.e., develop suitable analysis tools and corresponding analysis databases, through driven meetings within the team (recommendations for analysis tools and techniques are provided under the adapted Deliver phase). Traditional qualitative inquiry normally favoured in academic literature involves data interpretation procedures that are used to ensure the robustness and validity of study findings, but these procedures are time-consuming and therefore costly (80). Taking a design research perspective means adopting a much faster approach to understanding and reconciling competing requirements. Product development is part of a larger iterative learning process, where conclusive academic accuracy is not the primary end goal. Rather, it is a process of refining and understanding the users’ needs and aspirations and how they might be implemented in response (70). Accordingly, we recommend choosing faster and more agile data analysis and decision-making tools which allow for design errors and learnings to be captured at each step of the iteration, as assumptions about the product requirements are either challenged or validated until consensus on a final design solution is reached.

### Adapted develop phase

We propose that the adapted Develop phase include conducting debrief meetings online to go through completed activity packs with the end-users and allow for discussion and elaboration around responses. This involves keeping standard face-to-face procedures such as briefing, sending study materials and information sheets ahead of time and using a semi-structured interview format but adapting these so they can be done remotely. We suggest that interviews are recorded (in line with data protection regulations and best practices) and data transcription along with processing and reviewing the returned activity pack. Again, before formal analysis, it is suggested to bring unexpected findings to advisory groups at this stage to allow for exploration of rarer ones and allow for live discussion between stakeholders. By the end of this stage, the activity pack, verbatim interviews, and transcriptions should be completed.

### Adapted deliver phase

#### Data analysis

This phase should include data analysis to define needs and user priorities. Here, we describe specific data analysis tools. We suggest using Rapid Qualitative Inquiry as the means to facilitate credible qualitative research under limited time conditions. Rapid Qualitative Inquiry takes an intensive, diverse, team-based approach to the qualitative exploration of end-user perspectives, incorporating triangulation, iterative data analysis and supplementary data collection techniques to quickly establish a focused understanding of situations, experiences, or practices (81). Rapid Qualitative Inquiry requires at least two individuals to complete and can produce results in as little as five days, though it can often take several weeks. By drawing on the experience and perspective of individuals with different theoretical, backgrounds, disciplines and research skills, this approach is thought to substantially minimise the time required to collect and analyse data to gain sufficient knowledge of the topic area of interest.

We suggest that researchers go through the transcriptions and verbatim data, highlight important findings, and perform online sorting and grouping exercises as a proxy for commonly used in-person design research organisational tools such as post-it note sorting. We suggest developing a data matrix containing inductively generated priorities and separating those into negatively and positively valenced columns with supporting quotes mapped to the needs, like traditional qualitative research. Major learnings should include a full list of design needs and preferences, which can be used to categorise this information according to a novel data analysis tool informed by design principles, the design needs hierarchy. From the bottom up, this model depicts basic to more complex design priorities. Specifically, we propose four layers; 1) function - does the product fulfil its base function?, 2) usability - are the target end-users happy to use this device? 3) value - does the product enhance the end-user’s life in some way? and 4) delight - does the product exceed end-users expectations?

To complement participant-generated data, we advise the core team to perform a design feature selection analysis. This technique involves reviewing existing exemplars of the intended product to determine which features, if any, from already available designs could satisfy the cohort’s requirements. These features can then be ranked on a dimensional scale for suitability according to the design needs hierarchy requirements and used to come to a consensus about the best possible solution for each required attribute.

#### Prototyping

Finally, data generated at this stage should be used to model a minimum viable product (MVP), which is a validated, early solution prototype comprising sufficient features to be just agreeable to the target user group. The resulting MVP should aim to incorporate as many of the requirements from the design needs hierarchy, comprising the top-scoring items design feature selection analysis where applicable or novel design features where necessary. Here, we recommend enlisting the advisory group for feedback on these designs to inform progressive MVPs. As a result of this process, research teams should have a detailed specification of the MVP as well as a list of the materials, resources or procedures required to facilitate an MVP.

### PACES operationalisation

We set out to answer the problem statement ‘How do you conduct remote research with neurodivergent paediatric groups?’. We defined the research landscape through desk research and advisory discussions with families, teachers, and clinicians to determine the topic areas we needed to explore with the participants, for example, understanding home and school life, preferences for materials, functionality. Following this, we adopted an iterative design approach to refine an online interview strategy and activity pack through co-design with individual caregivers, sequentially incorporating their feedback. This reciprocal process was repeated three times, ensuring the final research processes and materials reflected our user group’s needs. Below, we discuss key outputs and learnings from applying our revised divergent-convergent framework.

#### Online interviews

In the absence of face-to-face introductions and ‘ice-breaker’ games, we surmised that the virtual nature of online workshops may make it harder for children with ADHD to express their thoughts and feelings in front of multiple participants simultaneously, which could stifle valuable information sharing. For this reason, in consultation with the ADHD advisory group, we decided to conduct user research sessions on an individual basis. We found this to be most effective for our project, but both individual and group sessions hold value and should be considered based on project-specific context and aims.

Research using varied methods has shown that children are reliable informants, capable of providing insightful and useful information if the correct methods to voice their thoughts and ideas are used (82, 83). Recommendations for conducting traditional semi-structured interviews with children on the younger end of our cohort (≤ six years old) outline that less structured methods should be employed during research activities. Furthermore, evidence suggests that referential communication (i.e., describing an object to another) is possible from the age of four or five years, allowing that the reference object is familiar, and the face-to-face interaction is conducted under familiar and naturalistic conditions (i.e., their home) (84). Building on the adapted D4D for ADHD framework, we employed several methodological adaptations to tailor one-to-one online interviews with neurodiverse children.

Caregivers were identified as crucial co-facilitators who helped children access the technology needed to participate in the online interviews, and provided in-person support for their child during the design process (including completing the activity pack). Their unique understanding of their child helped children to express themselves; they also provided additional collaborative insights, and motivated and kept their child on task, or in some cases scribed for their child (44, 85, 86). Caregivers also recommended that there be no more than two members of the research team facilitating the interview, to avoid overwhelming the child. In this regard, their presence was thought to be a source of familiarity for children and help support equitable power interactions between researchers and child participants, which has been identified as an important factor in positive co-design experiences with children, to ensure they feel like ideas and opinions are being taken seriously (87).

Consulting with the ADHD advisory group after conducting interviews was essential to convey ‘key lessons’ learnt and to sense-check decision-making. Like ‘member checking’ in traditional qualitative research (88–90), this consultation allows for examining the validity of findings at each stage and assessing accuracy and alignment with the lived experiences of the advisory group. It also allows for exploration around the relevance and weight of observed learnings compared to others. Furthermore, it allows for discussion of rarer findings, which is especially important to ensure the product or intervention is as inclusive as possible. Finally, due to the limitations of the individual interviews, the consultations provided an opportunity for discussion between stakeholders.

Families of children with ADHD (and similarly with other physical or mental health conditions) already experience a high burden and extraneous pressures associated with their diagnosis. Therefore, every effort should be made to minimise participant burden by scheduling interviews when it best suits families and couriering any physical materials in advance to families’ preferred addresses. Regarding the modality of data collection, caregivers advocated for varied information-gathering options to ensure activities were accessible to all children, enabling children to participate within their abilities. It is very common for children with ADHD to have a comorbid condition; evidence suggests that approximately 80% of children will have at least one additional diagnosis (91), including autism spectrum disorder (92), and developmental coordination disorder/dyspraxia (93), often presenting difficulties with social interaction and communication and coordination and motor programming respectively (93, 94). Therefore, to optimise verbal and written communication, we chose the activity pack as our primary source of data collection supplemented by verbal elaboration on the workbook answers during online debrief sessions with the design team. Importantly, we provided families with the choice of receiving a paper or electronic version of the activity pack, along with a stylus for children who opted to annotate the pack electronically.

The interviews needed to account for the different attention spans and distractibility inherent to ADHD (94). Children with ADHD often have difficulties sustaining concentration, and concurrent diagnoses like ASD can present further complications in conversation and social comprehension to a greater extent than typically developing children, which may be problematic for research that relies on structured interviews (95). Therefore, a successful co-design environment requires substantial flexibility, with the length and pace of online meetings dictated by participants, allowing them sufficient freedom to leave and rejoin sessions. Furthermore, to limit exposure to content that would be less interesting to children, caregivers suggested splitting the interview stage of this research over two sessions, whereby, caregivers were invited to the first session which involves administrative tasks, such as providing information sheets, consent and child assent forms, and instructions for completing the activity pack. The child would then only attend the second session accompanied by the caregiver(s) and only talk through the activity pack and be probed on their provided responses. Families were given a choice regarding the order in which they completed the activity pack activities, in line with their child’s preferences and the length of time needed to complete the activity pack, which varied considerably depending on how long children could attend to completing the pack. Similarly, the structure of the second family interview was directed by discussing the topics the child found most pleasing first, for example many children opted to begin the interview talking to us about the activity band they designed first, which was the penultimate task in the activity pack.

The co-design interview process highlighted that good documentation and communication were essential. All researchers assumed responsibility for taking notes, including visual cues, and memos i.e., internal reflections; communication between researchers using a private chat function provided immediate feedback to springboard further probing. The PACES interviewing team also met independently after each interview session to debrief, expand, and electronically document their reflection logs, so this information could be used iteratively to guide future interview cycles and research materials i.e., the activity pack.

#### Activity pack

The purpose of our activity pack was to replicate creative participation in a remote space in a sustaining and engaging manner; as such, it comprised varied and fun data collection options which were age- and ability-appropriate. For example, activities included a mixture of response types, including writing, drawing, multiple choice selection, card sorting, drawing pictures, and taking pictures, though families were advised that these response modalities were simply advisory, and children could complete the pack as they saw fit, for example writing instead of drawing or leaving the activity blank and verbally responding. Equally caregivers were permitted to scribe for their child if none of the advised data collection modalities were suitable. Greater weighting was given to visual methods, as research suggests these approaches may give rise to more varied and detailed responses compared to those that rely solely on verbal or written expression (96, 97). Each section included pages to be completed by both the child and their caregivers (differentiated by their colour, tone of voice, and instruction complexity), to gather both their perspectives and consolidate child responses. Additionally, large paper size was used to enable free expression via doodling, as well as using large clear fonts and printing on coloured stock.

**Figure 4:**
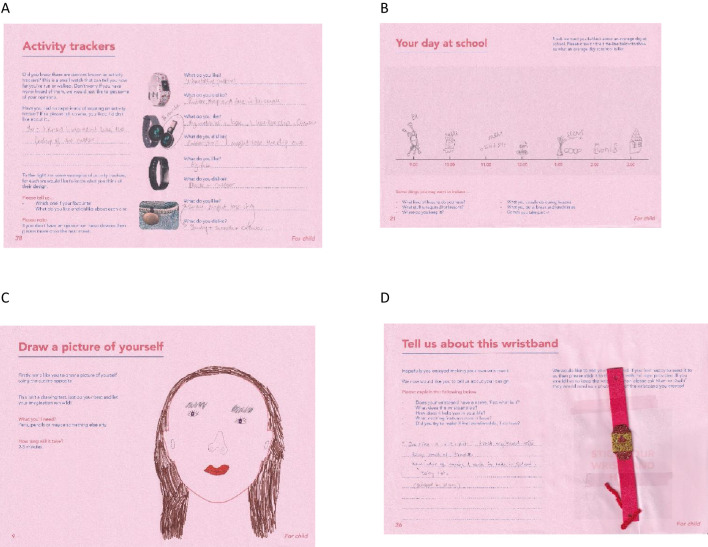
Examples of activities from the activity pack including, **(A)** documenting their preferences towards existing activity tracker typologies, **(B)** documenting their day, **(C)** drawing a picture of themselves, and **(D)** children crafting an activity tracker.

In terms of content, the activity pack focused on collecting information about participant demographics, interests, belongings, and daily routines to understand environments where the final design solution may be used and to identify how it can best fit into participants’ reality. We also developed exercises to gain an in-depth exploration of participants’ thoughts, feelings and needs about the end product. This included providing participants with first hand experience of the future device to prospectively assess their likes and dislikes; in our case, we asked children to wear a generic silicone wristband for 48 hours as a representation of the final wearable device and asked them to record how it made them feel both physically and emotionally. Additionally, we provided crafting materials and asked participants to design their ideal version of the end product to provide aesthetic design inspiration but also to understand children’s sensory needs by capturing their motivation for selecting materials, shapes, or sizes. Finally, we attempted to capture participants’ imagined or real experiences with similar products by asking them to appraise existing product archetypes and think about how interacting with the resulting end-product would make them feel, as well as asking about prior personal knowledge or involvement with comparable wearable devices. We encourage this activity where applicable to individual projects. **Figures 4A–D** provides an illustrative example of completed activity pack activities.

#### Decision-making processes

**Figure 5:**
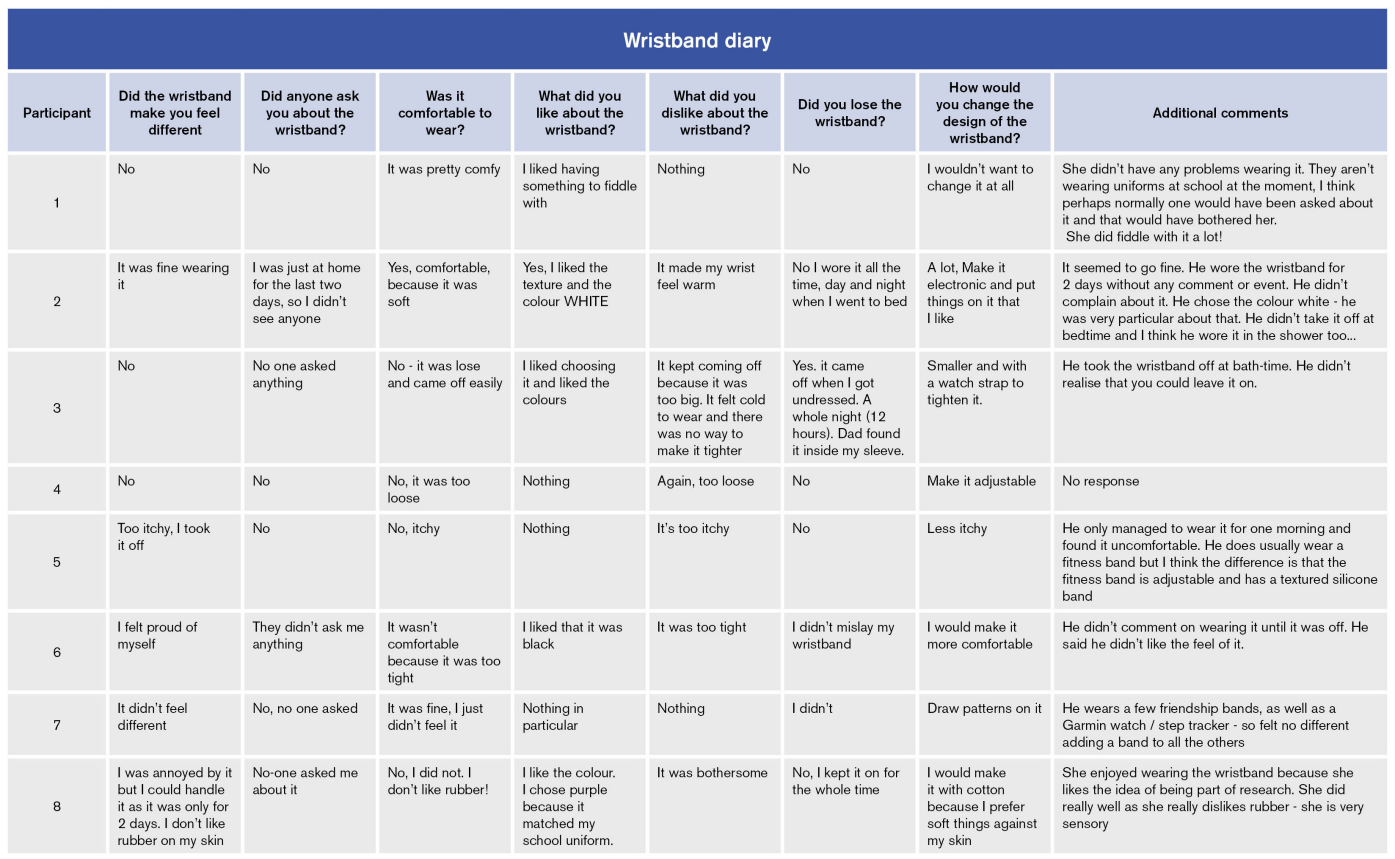
Example of how activity pack verbatim and paper-based responses were organised to facilitate analysis.

Following the RQI steps described above (81), data organisation was carried out to enable speedy decision-making and establish a series of design priorities by reconciling conflicting data points: namely, what co-designers needed versus what they wanted in the context of what was achievable within the constraints of the project, i.e., cost, time, essential functionality, technical feasibility, and safety. **Figure 5** provides an example of how verbatim and paper-based responses were organised to facilitate design prioritisation.

**Figure 6:**
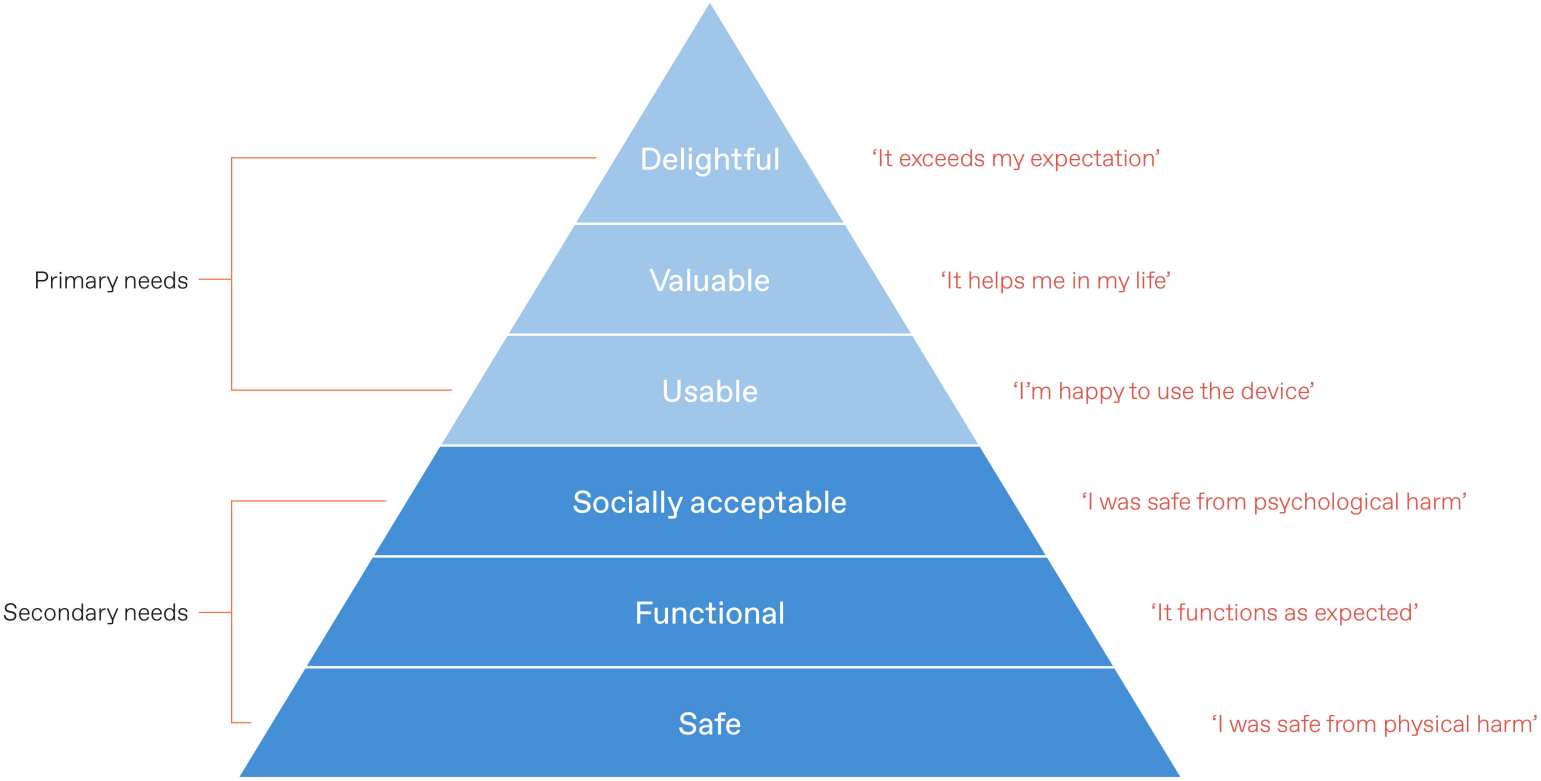
Overview of the revised design needs hierarchy.

We arranged learnings according to the design needs hierarchy. For this project, we chose to modify the model to accommodate two important emergent themes: safety – does the device pose any psychological or physical harm to the end-user?, and social acceptability – does the device limit the likelihood of device-related bullying? **Figure 6** provides a visual depiction of the revised design needs hierarchy. This data logic tool helped to decide upon the inclusion of user requirements necessary for the first MVP. This involved maximising the highest user priorities across all domains, while needs which took lower precedence were highlighted as potential attributes to be addressed later in the MVP roadmap.

**Figure 7:**
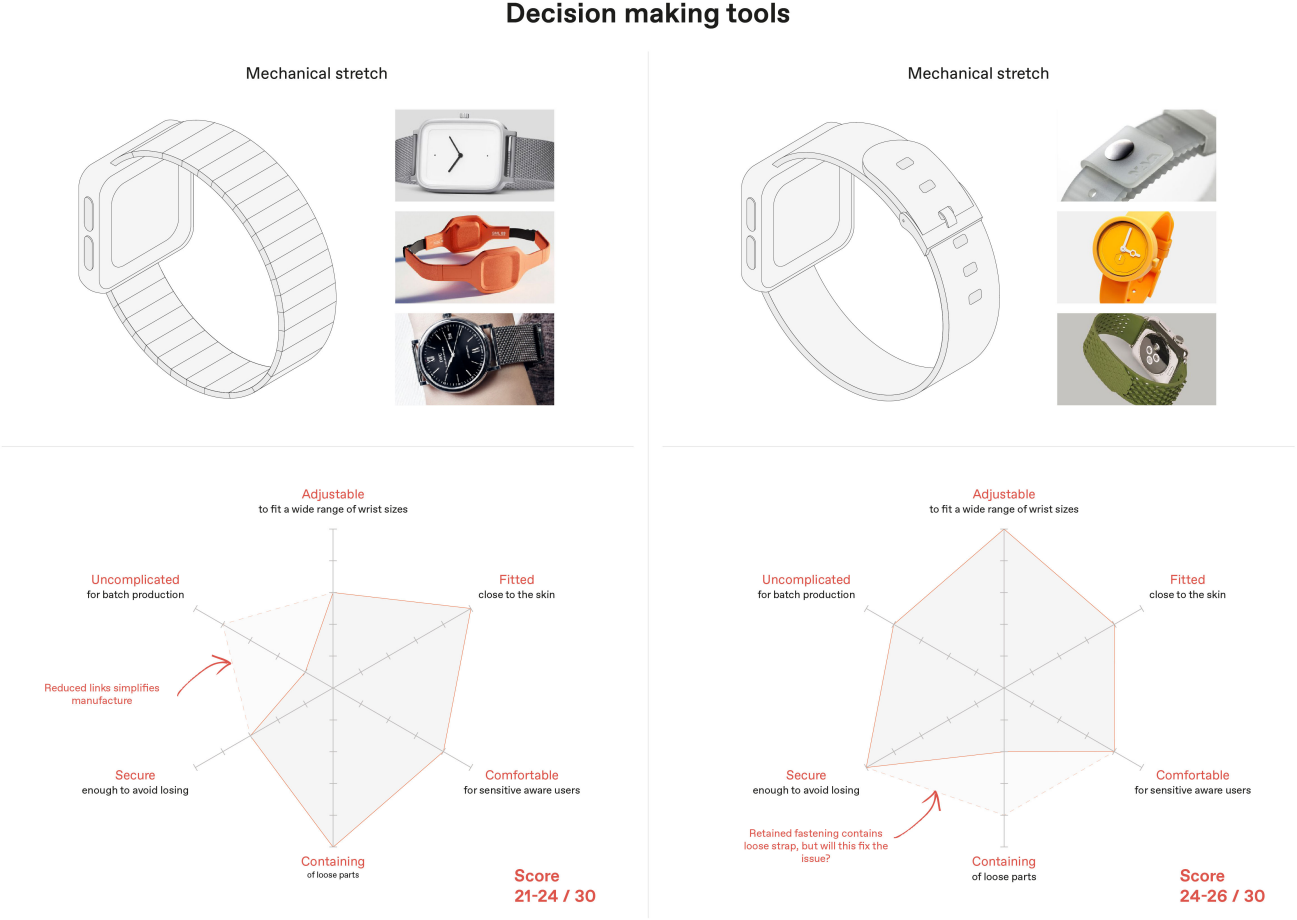
Scored examples of wristband strap typologies derived from design features analysis.

Data from the design feature analysis was then used to model Computer Aided Designs and create simple physical approximations – i.e., rapid prototypes of wrist-band models with different attributes. These attributes were ranked for suitability on a product radar chart according to different dimensions which emerged as categories from co-designer feedback and defined design constraints. This technique was developed to objectively inform the decision-making process rather than relying solely on designers’ experience, thus, enabling non-design core team members to be proactively involved. **Figure 7** demonstrates the comparison between possible wristband strap typology scores to allow for transparent decision-making to be fed back to families for sense-checking.

**Figure 8:**
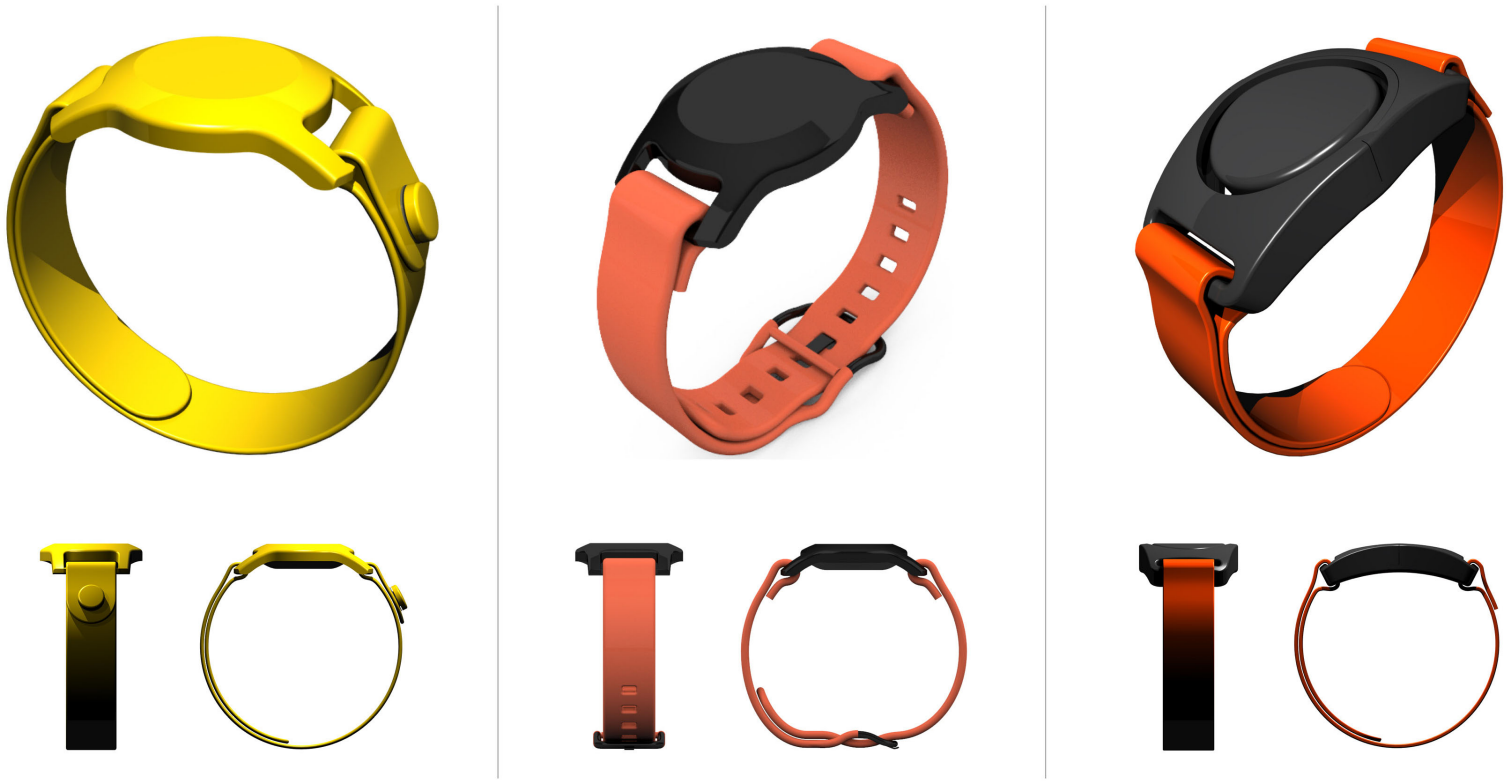
Example of three early minimum viable product (MVP) activity trackers.

After establishing MVP, early digital renderings of the PACES device were brought back to the ADHD advisory group to ensure the prototype aligned with the feedback they had previously given. **Figure 8** depicts an example of early digitalised MVPs shared with the advisory families. The main purpose of this exercise was to confirm with advisors that we interpreted our findings correctly. This MVP user feedback validation loop is an essential component of agile design which enabled us to iteratively develop the PACES device generated from stakeholder-reported information.

### Resulting overarching principles

- Utilise remote technologies such as phone calls and video conferencing, choosing the platform based on researcher and user needs and take advantage of conducting research in a home environment to increase research accessibility for harder to reach groups.
- Include caregivers as key co-researchers ensures equitable power distribution, and familiarity, supports accurate articulation, and helps maintain concentration.
- Incorporate both individual sessions and group sessions, while sense-checking individual interview findings and potential design solutions with the advisory group at each stage.
- Employ ongoing communication between all stakeholders.
- Be closely familiar with neurodiverse needs before commencing the design process to engage end-users.
- Adopt a flexible, agile, adaptive, and creative approach, allowing child-caregiver dyads to choose the preferred time, pace, length, and order of activities, thus, catering to their needs, minimising time burden, and maximising inclusion.
- Utilise diverse and creative methods for data collection (e.g., combined paper/online modalities with a variety of tasks and activities).
- Employ iterative processes in each stage, to successfully prioritise competing views, and arrive at decisions and solutions collaboratively.
- Be transparent in design decisions – multidimensional assessment and decision reconciliation of needs versus limitations.

## Discussion

Co-design is widely recommended in the healthcare design space as a method to develop an empathic understanding of patients and produce design solutions that meet specific user needs. However, co-design has been criticised for its lack of methods codified in the literature, with a particular paucity of research describing or evaluating these approaches in detail (15, 34). Moreover, it is commonplace in co-design practice for designers to either define their methods based on experience or use those they have learned from other designers, further contributing to its elusive nature. While there are examples of successful user-led healthcare product development in the literature (98), there is less guidance available regarding how to successfully co-design with ADHD populations. Shifting to remote research in response to the COVID-19 pandemic has added yet more complexity and a need for further adapted guidance.

This paper contributes to the methodological evidence base by offering combined collaborative experience and a flexible approach for conducting remotely situated design processes and decision-making, adapted to the idiosyncrasies of target stakeholder groups to ensure suitable design tools and activities are used. It provides step-by-step actionable insights as well as a repository of transparent, understandable, and goal-orientated tools and guidance on how and when to deploy them based on our experience co-designing with neurodiverse children. Additionally, our work provides insight into how qualitative user research methods - which are increasingly favoured by healthcare providers for product innovation, including the NHS - can be applied rapidly (99). Through the culmination and application of design-informed data analysis processes in FREDY i.e., design needs hierarchy, design feature selection, and sharing digitalised prototypes with the ADHD caregivers’ group at each stage of the co-design process to accrue immediate feedback, we demonstrate how to quickly structure competing priorities while prioritising consensus decision-making.

## Strengths and limitations

Our findings suggest that replacing shared group activities with independently completed workbooks allowed children to communicate their ideas through multiple modalities at their own pace, which may have enhanced active participation and improved the quality of the resulting data. This approach also likely broadened the co-design process to populations who may struggle to engage with traditionally structured workshops due to challenges with their social, emotional, cognitive, linguistic or mobility skills (37). We further postulate that this methodology could also be useful in future research projects exploring sensitive topics, where direct verbal disclosure may cause discomfort or important information to be withheld.

Applied more broadly, FREDY may have additional benefits. By reducing the need to travel to a physical location, this type of research may afford more inclusive sampling and greater socio-demographically diverse representation – i.e., participation from marginalised or excluded due to challenges of commuting, disability, social difficulties, childcare, or work schedules, who are often harder to reach. Their involvement identifies and gives credence to the needs of these populations that require product innovation and allows for meaningful input to decision-making processes that relate directly to them (37, 100). In our example, we were able to recruit teachers from urban and rural locations, reducing the impact of ‘distance decay’ a phenomenon whereby the likelihood of individuals participating in research reduces the greater distance to be travelled (42). Single-caregiver families without access to additional childcare resources and families with complex living arrangements (living between two different households in different parts of the country) who would not have participated under ordinary situated co-design circumstances were also able to take part (101).

We found that caregiver involvement as proxy researchers was vital to the success of the virtual workshops. Guidance relating to their child’s mood, working styles, and times of focus or productivity meant that interviews were uniquely tailored to each child, providing an extraordinary level of support, structure, and advocacy (102, 103). During debrief meetings, caregivers naturally managed their child’s behaviour, predominantly taking this responsibility away from the researchers; this likely minimised children’s perceptions of the adult researchers as authority figures and may have given rise to more transparent and honest responses. It is however equally possible that given their age and experience, children may have still told the researchers what they thought they wanted to hear (82). Of note, some caregivers said that completing the activity pack was valuable to their family, as it gave them new insights into their child’s life and condition. As such, we encourage other design and research teams to consider using this approach either independently or as part of a wider qualitative programme of work where in-person contact is permitted. Including this type of familial or live-in external support may also be beneficial to remote co-design with children more broadly, as well as with vulnerable adult populations i.e., the elderly and intellectually and/or physically disabled populations.

Conversely, relying on high levels of caregiver supervision may pose risks. Caregivers may unintentionally over-articulate or interject on their child’s behalf (44), distract their child (104) or the child may ‘caregiver-please’ as they are used to the caregiver being in charge (105) and thus stifle creative expression. This may be troublesome for design teams with tight deadlines as children may take a considerable amount of time to acclimatise to the change in power dynamics. Further power imbalances can ensue from increased reliance on caregivers as they typically control access to and operate the technology being used to host the co-design session (44). However, the benefits may outweigh the costs of caregiver involvement in the case of neurodiverse populations, where the prevalence of comorbid dyspraxia, is high which may affect digital competency. Moreover, depending on the nature of the co-design project, there may be topics children would prefer to discuss in private rather than with their caregivers. This is a sensitive area that will need to be considered carefully by the research team to ensure children get a chance to speak independently if appropriate.

While individualised remote co-design enables more personalised engagement, limitations include the total lack of in-person interaction, which can restrict rapport and trust between participants and researchers (44). Additionally, there are associated cost and time implications to consider; for example, whereas traditional co-design activities usually take place in a shared space with visual aids such as a whiteboard, FREDY requires research material including workbooks, crafting materials and styluses to be couriered to and from families (37).

Due to resource constraints, there was no formal evaluation of this framework, nor did we have the capacity to formally contrast it to another framework or traditional focus group work. Similarly, though we received verbal feedback from participants regarding the merits or weaknesses of this approach, we were unable to conduct a formal process evaluation with the co-designers and their families to understand their experience of taking part in virtually situated participatory research guided by this framework. While it would have been useful to see how FREDY performed in terms of continued conversation and feedback from stakeholders on physical rather than digital prototypes as well as the subsequent deployment phase of prototyping (106), it wasn’t possible due to the technical complexity of the end-product and manufacturing requirements.

Finally, in our example, we do not provide guidance on remote co-design for groups and advise that the very nature of the activity pack and the way it is used is inherently personal. Therefore, we encourage readers to explore other literature regarding simultaneous multiple stakeholder engagement in an online environment, including practical tips, such as selecting appropriate communication and collaborative platforms, and building and maintaining online group dynamics, and togetherness.

## Conclusion

The current paper adds to the co-design health literature by providing a new theoretically informed method for conducting co-design research with ADHD children under conditions necessitating remote engagement. Our framework promotes the use of cyclical iterative stages from the outset of a project to enable accelerated user-informed design responses. In FREDY, co-designer input is incorporated during the development and application of co-design materials and engagement procedures, as well as decision-making strategies, to ensure the entirety of the outcome of the co-design process fully encapsulates end-users’ needs and wants. This approach also offers a roadmap for future product enhancement beyond early prototyping, and the potential to minimise time and financial constraints which are often key barriers encountered in academic grant-funded work. While many of the techniques described in the paper are not novel, we demonstrate how existing processes can be combined for working with neurodiverse children and can be applied to co-create with this cohort remotely. We discuss the potential benefits of remote user research arising from interruptions to in-person workshops and demonstrate how the considered use of design principles and tools resulting from an interdisciplinary academic and industrial collaborative partnership allowed for quick design modifications not typically observed in the qualitative research space.

## Data Availability

The raw data supporting the conclusions of this article will be made available by the authors, without undue reservation.
