# Supplementary figures and images for: A framework for remotely enabled co-design with young people: its development and application with neurodiverse children and their caregivers
Source: Front Psychiatry. 2024 Aug 16;15:1432620. doi: 10.3389/fpsyt.2024.1432620 (PMC11362057; doi:10.3389/fpsyt.2024.1432620)

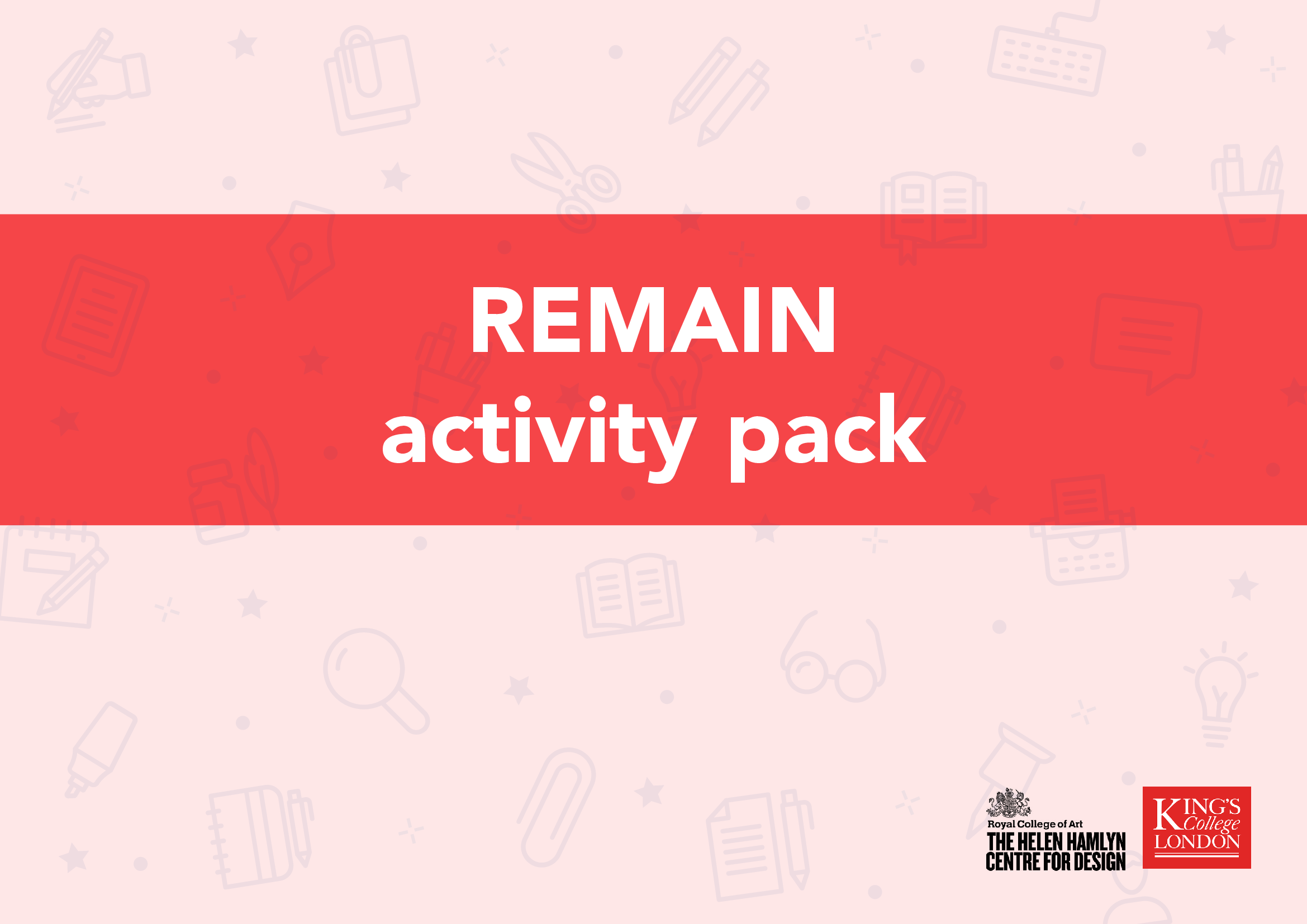

Supplement: Supplementary file 1 [file Image1.tiff]
